# Pinhole Effect on the Melting Behavior of Ag@Al_2_O_3_ SERS Substrates

**DOI:** 10.1186/s11671-016-1390-0

**Published:** 2016-03-31

**Authors:** Lingwei Ma, Yu Huang, Mengjing Hou, Jianghao Li, Zhengjun Zhang

**Affiliations:** State Key Laboratory of New Ceramics and Fine Processing, School of Materials Science and Engineering, Tsinghua University, Beijing, 100084 People’s Republic of China; Key Laboratory of Advanced Materials (MOE), School of Materials Science and Engineering, Tsinghua University, Beijing, 100084 People’s Republic of China

**Keywords:** Metal@oxide nanostructures, Pinhole shell, Thermal stability, Melting point, SERS

## Abstract

High-temperature surface-enhanced Raman scattering (SERS) sensing is significant for practical detections, and pinhole-containing (PC) metal@oxide structures possessing both enhanced thermal stability and superior SERS sensitivity are served as promising SERS sensors at extreme sensing conditions. Through tuning the Al_2_O_3_ precursors’ exposure time during atomic layer deposition (ALD), Al_2_O_3_ shells with different amount of pinholes were covered over Ag nanorods (Ag NRs). By virtue of these unique PC Ag@Al_2_O_3_ nanostructures, herein we provide an excellent platform to investigate the relationship between the pinhole rate of Al_2_O_3_ shells and the melting behavior, high-temperature SERS performances of these core-shell nanostructures. Pinhole effect on the melting procedures of PC Ag@Al_2_O_3_ substrates was characterized in situ via their reflectivity variations during heating, and the specific melting point was quantitatively estimated. It is found that the melting point of PC Ag@Al_2_O_3_ raised along with the decrement of pinhole rate, and substrates with less pinholes exhibited better thermal stability but sacrificed SERS efficiency. This work achieved highly reliable and precise control of the pinholes over Al_2_O_3_ shells, offering sensitive SERS substrates with intensified thermal stability and superior SERS performances at extreme sensing conditions.

**Electronic supplementary material:**

The online version of this article (doi:10.1186/s11671-016-1390-0) contains supplementary material, which is available to authorized users.

## Background

High-temperature surface-enhanced Raman scattering (SERS) detection is a vital part for practical sensing, which can be employed for monitoring many in situ reactions, e.g., thermal crystallization [1], structural variations [2, 3], and chemical reactions [4, 5] at elevated temperatures. However, bare metal nanostructures suffer from the inherently low melting point [6, 7], which causes the morphological instability of nano-sized metal and as a result, may deteriorate their SERS performances at high temperatures [2–4, 8–10]. Lately, core-shell nanostructures of metal core covered with protective oxide layer have been proposed as good SERS substrates for high-temperature Raman sensing [2, 3, 5, 8–10]. For example, wrapping Ag nanorods (Ag NRs) with an ultrathin (~1.5 nm) but dense Al_2_O_3_ layer could make the substrate robust in morphology at 400 °C and stabilize its SERS efficiency [10]. Most recently, novel metal@oxide structures with pinhole-containing (PC) shells have drawn tremendous attention, which could not only increase the working temperature of SERS substrates moderately [10], but also exhibited even better SERS properties and broader application fields compared with metal@oxide substrates of compact shells [11, 12]. Accordingly, it is highly desired to synthesize PC metal@oxide structures as ideal SERS-active substrates as well as investigate and optimize their properties.

However, up to now, the accurate control and measurements of the oxide pinhole rate, as well as the comprehensive investigation of the melting procedures and thermal stability of PC metal@oxide substrates, have not been investigated in detail. In this regard, herein we introduced atomic layer deposition (ALD) technique to cover Ag NRs with Al_2_O_3_ shells (Ag@Al_2_O_3_) of different pinhole amount and experimentally analyzed the Al_2_O_3_ pinholes’ influence on the melting behavior of PC Ag@Al_2_O_3_ substrates. The pinholes can be readily tuned by varying the exposure time of Al_2_O_3_ precursors during ALD coating, and the pinhole rate was estimated using the Raman signals of acridine molecules on uncoated Ag NRs and PC Ag@Al_2_O_3_ substrates. The melting process of PC Ag@Al_2_O_3_ was monitored via their reflectivity variations during heating, and the melting point of different substrates was quantitatively calculated and compared. In addition, the SERS performances of PC Ag@Al_2_O_3_ substrates were tested after thermal treatment, demonstrating excellent stability and versatility of these SERS sensors.

## Methods

### Fabrication of Ag NRs

Slanted Ag NRs were prepared on Si (001) substrates by oblique angle deposition (OAD) technique in an electron-beam system (GLAD, Thermionics Inc.) with a background vacuum level of 10^−6^ Pa. During deposition, the incident angle between the surface normal of substrates and vapor flux was set at ~86°, with a deposition rate of ~0.75 nm/s. The NR growth finished at a thickness of 1000 nm read by a quartz crystal microbalance [10, 12].

### Fabrication of PC Ag@Al_2_O_3_ Substrates

Al_2_O_3_ layers were coated onto the as-prepared Ag NRs in an ALD reactor (MNT-100, Wuxi MNT Micro and Nanotech Co.) at 50 °C. The Al_2_O_3_ precursors, i.e., trimethylaluminum (TMA; maintained at 150 °C) and water (maintained at 40 °C), were alternatively pumped into the reaction chamber using high purity N_2_ (99.999 %, 15 sccm) as the carrier and purge gas. In order to synthesize Al_2_O_3_ shells with a different pinhole rate, only one ALD cycle was used on top of Ag NRs and the exposure time of TMA and water was simultaneously changed during coating [10, 12]. One complete reaction consisted of four steps: (1) TMA reactant exposure, 2/5/10/20/40/80/100 ms; (2) N_2_ gas purging, 10 s; (3) water vapor exposure, 1/2/5/10/20/40/50 ms; and (4) N_2_ gas purging, 20 s. These substrates are denoted hereafter as Ag@Al_2_O_3_/2, Ag@Al_2_O_3_/5, Ag@Al_2_O_3_/10, Ag@Al_2_O_3_/20, Ag@Al_2_O_3_/40, Ag@Al_2_O_3_/80, and Ag@Al_2_O_3_/100, respectively. (These numbers represent the TMA exposure time during ALD coating)

### Characterization of Ag NRs@Al_2_O_3_

The morphology and structures of Ag NRs and Al_2_O_3_ shells were characterized by scanning electron microscope (SEM; JEOL-JMS-7001F) and high-resolution transmission electron microscope (HRTEM; JEOL-2011). The melting process of these substrates was monitored in situ via their reflectivity variations upon annealing, using Optical Power Thermal Analyzer (OPA-1200).

### SERS Detections

Acridine and 4-mercaptobenzoic acid (4-MBA) with different concentrations were dissolved into ethanol. SERS measurements were conducted by an optical fiber micro-Raman system (i-Raman Plus, B&W TEK Inc.). Before detection, all substrates were merged into different solutions for 1 h, washed thoroughly to remove the residual molecules, and dried naturally in air. Raman spectra were obtained using a 785-nm laser as the excitation source, with an excitation power of 150 mW and the data collection time of 10 s for each spectrum. For every sample, the spectrum was obtained by averaging the spectra obtained from five different areas of the SERS substrate.

## Results and Discussion

**Fig. 1:**
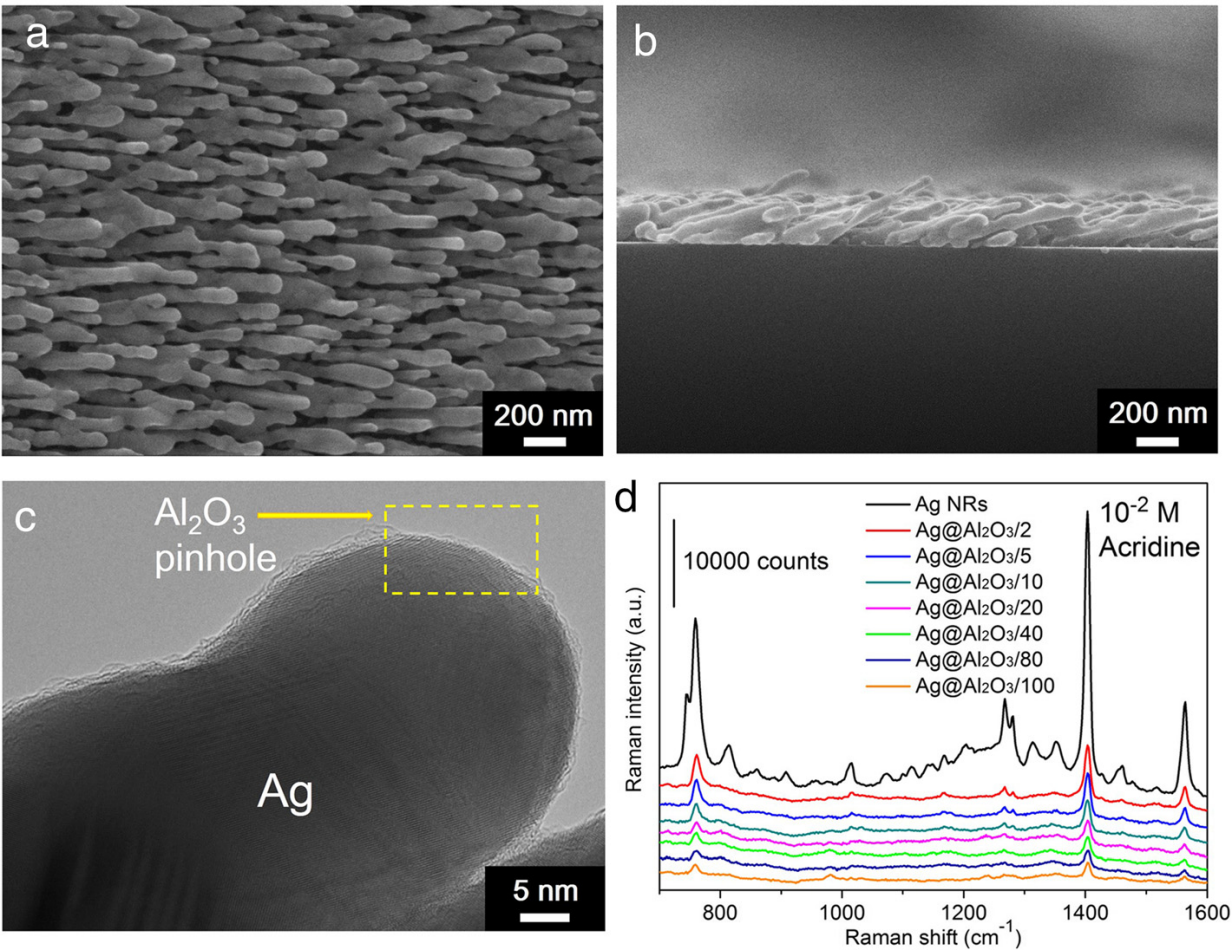
**a** Typical top-view SEM, **b** side-view SEM, and **c** HRTEM images of Ag@Al_2_O_3_/10 substrate. **d** Raman spectra of 1 × 10^−2^ M acridine from uncoated Ag NRs and PC Ag@Al_2_O_3_ substrates with distinct TMA/water exposure time

Figure 1a, b shows typical top-view and side-view SEM images of Ag@Al_2_O_3_/10 substrate, from which one sees clearly that the slanted NRs are well-separated and ~700 nm in length. Figure 1c illustrates the HRTEM image of Ag@Al_2_O_3_/10 with a PC Al_2_O_3_ shell, which is sub-nanometer thick and uniformly wraps Ag NRs. To explore the relationship between the exposure time of ALD precursors and Al_2_O_3_ pinhole rate, we introduced acridine, a SERS probe molecule that can directly interact with Ag surface instead of Al_2_O_3_ layers [11–13]. If there are any pinholes in Al_2_O_3_ shells, acridine would adsorb on Ag surface through pinholes and exhibit Raman signals, paving a reliable way toward the characterization of Al_2_O_3_ pinholes. One sees from Fig. 1d that the Raman spectra of 1 × 10^−2^ M acridine molecules [14, 15] showed up not only on uncoated Ag NRs but also on PC Ag@Al_2_O_3_ substrates with distinct TMA/water exposure time, indicating that all these substrates had PC shells with exposed Ag surface inside. Because of the saturation of 1 × 10^−2^ M acridine over uncoated Ag NRs (see Additional file 1: Figure S1), we further utilized the acridine spectra from different substrates to estimate the pinhole rate of Al_2_O_3_ shells, via dividing acridine Raman intensity at 1403 cm^−1^ from PC Ag@Al_2_O_3_ substrates by that from uncoated Ag NRs. It is shown in Table 1 that the Al_2_O_3_ pinhole rate declined gradually with the increment of TMA/water exposure time, suggesting that longer exposure time provide better opportunity for TMA and water to react over Ag NRs. The pinhole rate ranges from ~18.0 to ~5.3 %, and further longer exposure time results in no obvious change of the pinhole rate.

**Table 1:** Pinhole rate of different PC Ag@Al_2_O_3_ substrates

Sample name	Pinhole rate (%)
Ag NRs	100
Ag@Al_2_O_3_/2	18.0
Ag@Al_2_O_3_/5	14.9
Ag@Al_2_O_3_/10	10.7
Ag@Al_2_O_3_/20	7.9
Ag@Al_2_O_3_/40	7.0
Ag@Al_2_O_3_/80	5.5
Ag@Al_2_O_3_/100	5.3

**Fig. 2:**
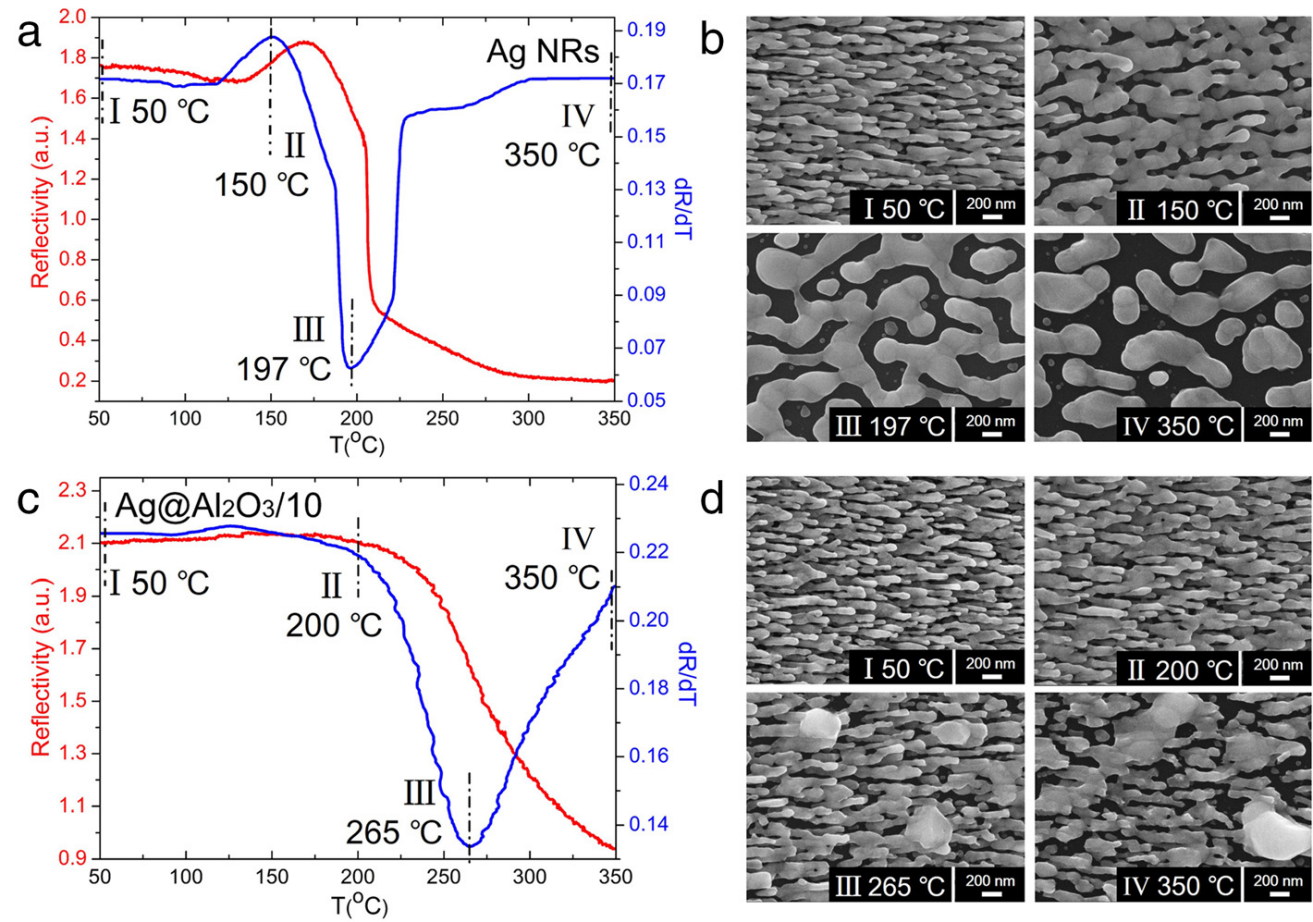
The melting procedures of **a** uncoated Ag NRs and **c** Ag@Al_2_O_3_/10 at 50–350 °C through monitoring their reflectivity changes upon annealing (*red line*), and the corresponding extreme point of the reflectivity’s derivative from each sample as their melting point (*blue line*). The morphological changes of **b** uncoated Ag NRs and **d** Ag@Al_2_O_3_/10 after heating

**Fig. 3:**
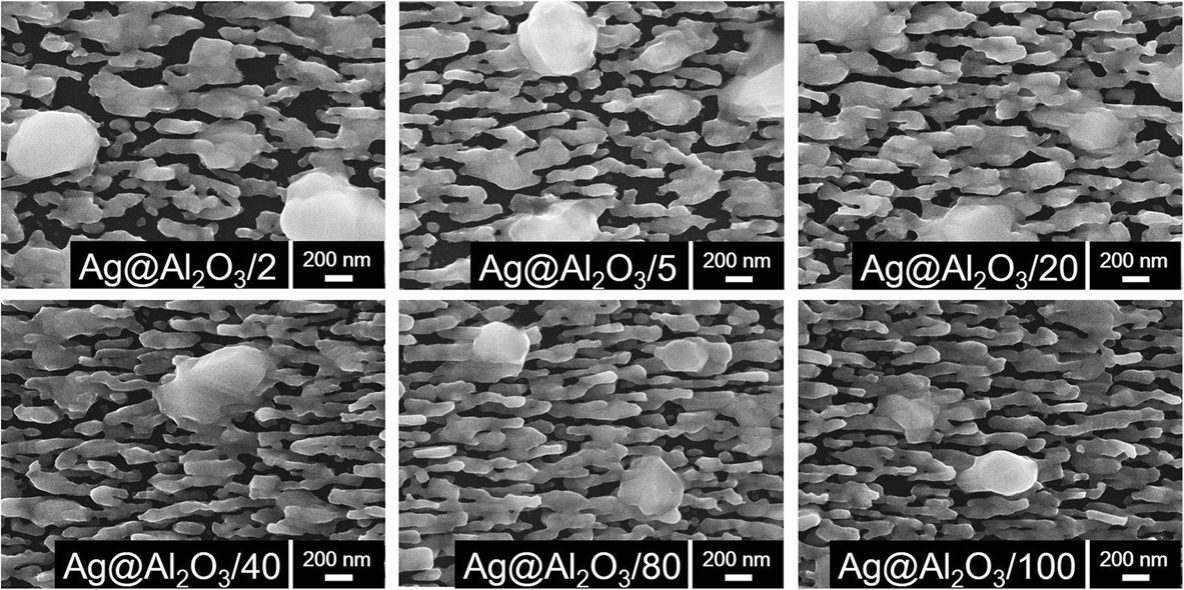
SEM images of Ag@Al_2_O_3_/2, Ag@Al_2_O_3_/5, Ag@Al_2_O_3_/20, Ag@Al_2_O_3_/40, Ag@Al_2_O_3_/80, and Ag@Al_2_O_3_/100 after heating at 350 °C.

**Fig. 4:**
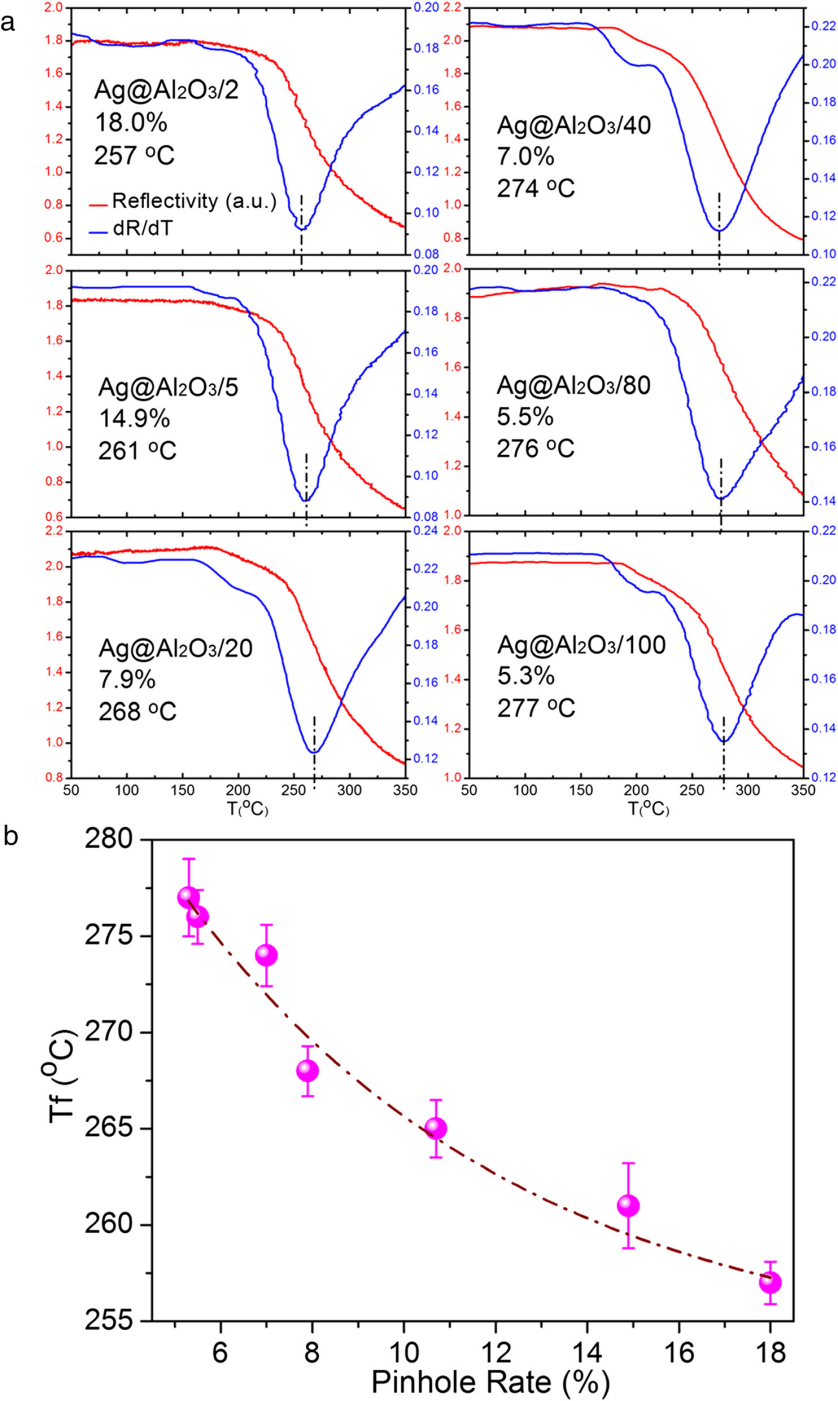
**a** The melting procedures and the extreme point of the reflectivity’s derivative from different PC Ag@Al_2_O_3_ substrates. **b** The melting point of PC Ag@Al_2_O_3_ substrates as a function of their pinhole rate

Since optical property of nanostructures is sensitive to their morphology [16, 17], the melting procedures of PC Ag@Al_2_O_3_ substrates can be characterized in situ via their reflectivity changes upon annealing. Figure 2 thoroughly investigates the melting process and morphological changes of uncoated Ag NRs and Ag@Al_2_O_3_/10 at 50–350 °C (red line). Because melting occurs during a continuous process instead of at a specific point, to specifically and quantitatively characterize the melting point of different substrates, we define the extreme point of the reflectivity’s derivative (blue line) from each sample as their melting point, at which temperature the morphology of nanostructures changes most dramatically [18, 19]. For bare Ag NRs, the reflectivity began to change at ~120 °C and the obvious shape variation was observed at 150 °C, which would affect the efficiency of SERS substrates. The structure distortion facilitated with increasing the annealing temperatures, and Ag NRs lost completely their shape after the melting point of ~197 °C, suggesting the instability nature of nano-sized Ag. As for Ag@Al_2_O_3_/10, the substrate maintained its shape at ~200 °C, and the melting point was as high as ~265 °C. It is particularly noted that although the morphology change of Ag@Al_2_O_3_/10 initiated at ~200 °C, the substrate kept partly the NR shape even at 350 °C, indicating the superior protection of Al_2_O_3_ shell. Figure 3 represents the SEM images of Ag@Al_2_O_3_/2, Ag@Al_2_O_3_/5, Ag@Al_2_O_3_/20, Ag@Al_2_O_3_/40, Ag@Al_2_O_3_/80, and Ag@Al_2_O_3_/100 after heating at 350 °C. It is observed that the substrates with less or smaller Al_2_O_3_ pinholes could keep better the nanorod shape and generate less fusion spots after annealing, indicating the pinhole rate’s effect on the thermal and morphological stability of these substrates. Furthermore, the melting point of various PC Ag@Al_2_O_3_ substrates as a function of their pinhole rate was quantitatively evaluated and depicted in Fig. 4. Remarkably, we found that their melting point increase monotonously with the decrease of pinhole rate, starting from 257 °C with ~18.0 % pinholes and reaching a maximum at 277 °C when the pinhole rate was ~5.3 %. These results clearly demonstrate that the Al_2_O_3_ coverage provides a useful opportunity to strengthen the thermal stability of Ag nanostructures, which also offers us a means to precisely control the melting point of PC Ag@Al_2_O_3_ substrates.

**Fig. 5:**
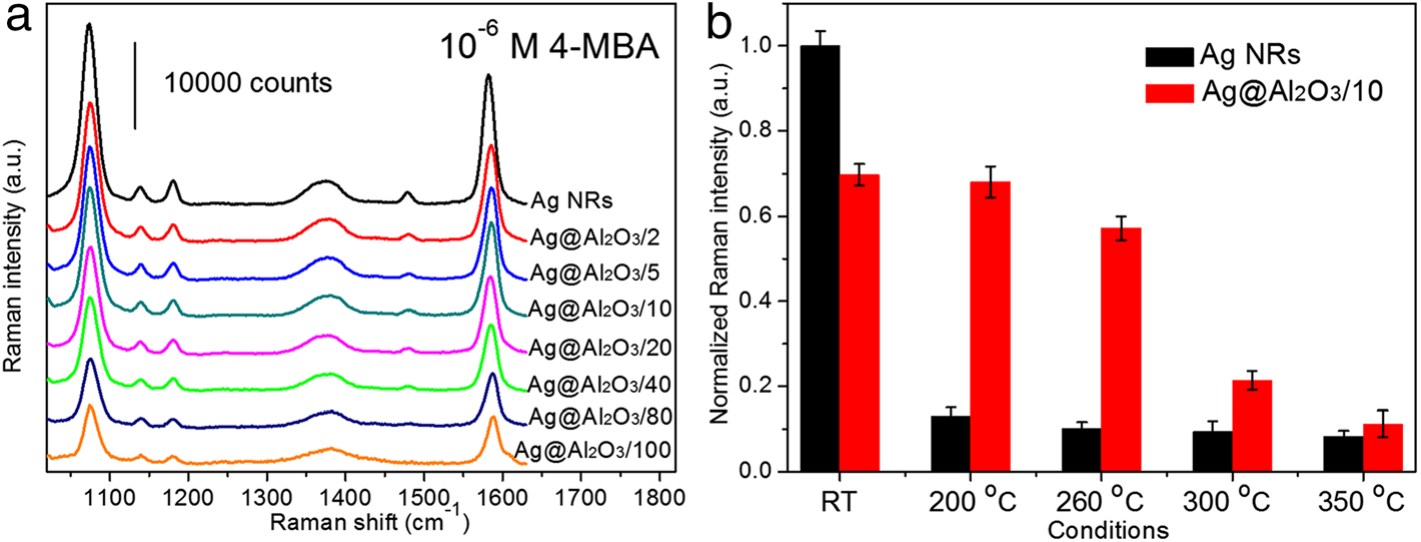
**a** Typical Raman signals of 1 × 10^−6^ M 4-MBA molecules from uncoated Ag NRs and distinct PC Ag@Al_2_O_3_ substrates. **b** Normalized Raman intensity of 1 × 10^−6^ M 4-MBA from uncoated Ag NRs and Ag@Al_2_O_3_/10 at various conditions, i.e., at room temperature (RT), and after annealing at 200, 260, 300, and 350 °C, respectively.

In the case of high-temperature SERS detection, 1 × 10^−6^ M 4-MBA [20, 21] was used to evaluate the SERS efficiency and thermal stability of PC Ag@Al_2_O_3_ substrates. Because SERS effect is highly localized and attenuates quickly away from metal surface [22, 23], a gradual decrement of 4-MBA Raman signals is observed with the decline of exposed Ag surface (see Fig. 5a). To be specific, the SERS intensity of 4-MBA on Ag@Al_2_O_3_/2, Ag@Al_2_O_3_/5, and Ag@Al_2_O_3_/10 with ~18.0 to ~10.7 % pinholes was about 80–70 % compared with that on uncoated Ag NRs. Further decrease of the pinhole rate led to a more dramatic drop of 4-MBA signals, which is consistent with the previous results of acridine molecules, except the fact that 4-MBA could interact not only with Ag surface but also with Al_2_O_3_ shells [24–27]. We should also mention that even though the SERS sensitivity of PC Ag@Al_2_O_3_ substrates decreased to some extent after ALD coating, all these substrates with ultrathin Al_2_O_3_ shells and pinholes were highly effective for trace analyte recognition [12].

To demonstrate the feasibility of PC Ag@Al_2_O_3_ substrates for real-world applications, Ag@Al_2_O_3_/10 combined with both high melting point and good SERS activity was chosen to assess its high-temperature SERS performances, utilizing uncoated Ag NRs as a reference. It is observed from Fig. 5b that, when bare Ag NRs were heated at 200–350 °C, due to their significant morphological changes, the SERS activity decreased about one order of magnitude. On the contrary, although, at room temperature (RT), the SERS intensity of 4-MBA on Ag@Al_2_O_3_/10 was ~70 % in comparison with that on uncoated Ag NRs, this substrate was extremely robust in SERS performance at elevated temperatures. Approximately 5.5 times higher SERS enhancement was obtained from Ag@Al_2_O_3_/10 compared with that from bare Ag NRs when heated at 200 and 260 °C, indicating the strongly improved SERS stability of PC Ag@Al_2_O_3_ substrates. At 300 and 350 °C, Ag@Al_2_O_3_/10 showed moderate declines in SERS signals, which can be explained by its structure changes when exceeding its melting point of ~265 °C.

## Conclusions

In summary, we successfully synthesized PC Ag@Al_2_O_3_ nanostructures with controllable pinhole rate and investigated in detail the relationship between the melting behavior of PC substrates and Al_2_O_3_ pinhole rate. Due to the unique structures of these substrates, the melting point of PC Ag@Al_2_O_3_ increased along with the decline of the Al_2_O_3_ pinhole rate. By coating protective Al_2_O_3_ layers over Ag NRs, the substrates could preserve their structures and SERS efficiency at temperatures higher than 250 °C. These PC Ag@Al_2_O_3_ substrates with a controllable pinhole rate exhibit great potential as advanced platforms for high-temperature SERS detections.

## Additional file

Additional file 1: Figure S1. Raman spectra of 1 × 10^−1^ M, 5 × 10^−2^ M, 1 × 10^−2^ M, and 1 × 10^−3^ M acridine molecules from uncoated Ag NRs. (DOCX 159 kb)

## Abbreviations

4-MBA: 4-mercaptobenzoic acid
Ag NRs: Ag nanorods
ALD: atomic layer deposition
HRTEM: high-resolution transmission electron microscope
OAD: oblique angle deposition
PC: pinhole-containing
RT: room temperature
SEM: scanning electron microscope
SERS: surface-enhanced Raman scattering
TMA: trimethylaluminum
