# Supplementary material for: Pinhole Effect on the Melting Behavior of Ag@Al2O3 SERS Substrates
Source: Nanoscale Res Lett. 2016 Mar 31;11:170. doi: 10.1186/s11671-016-1390-0 (PMC4816938; doi:10.1186/s11671-016-1390-0)
Supplement: Additional file 1: Figure S1. — Raman spectra of 1 × 10^−1^ M, 5 × 10^−2^ M, 1 × 10^−2^ M, and 1 × 10^−3^ M acridine molecules from uncoated Ag NRs. (DOCX 159 kb) [file 11671_2016_1390_MOESM1_ESM.docx]

Supporting Information

**Pinhole effect on the melting behavior of Ag@Al_2_O_3_ SERS substrates**

Lingwei Ma,^1^ Yu Huang,^1^ Mengjing Hou,^1^ Jianghao Li,^1^ and Zhengjun Zhang^2*^

- Correspondence: [zjzhang@tsinghua.edu.cn](mailto:zjzhang@tsinghua.edu.cn)

^1^ State Key Laboratory of New Ceramics and Fine Processing, School of Materials Science and Engineering, Tsinghua University, Beijing 100084, P.R. China

^2^ Key Laboratory of Advanced Materials (MOE), School of Materials Science and Engineering, Tsinghua University, Beijing 100084, P.R. China


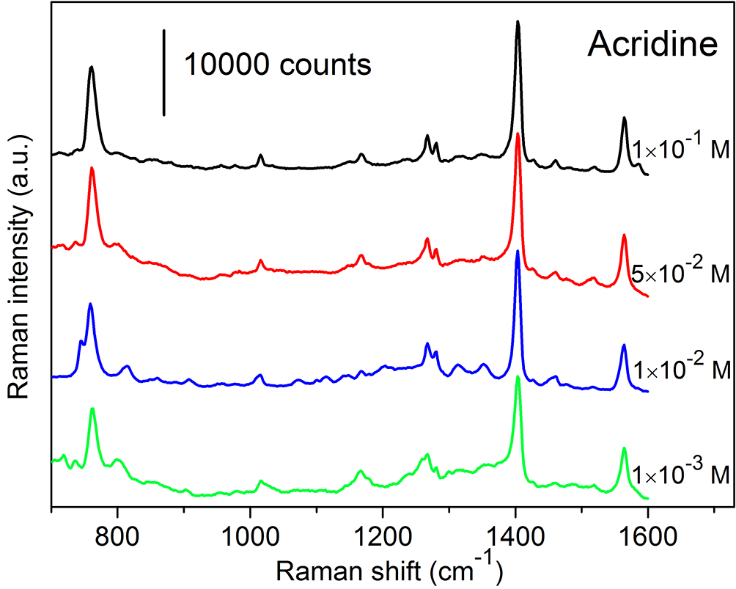


**Fig. S1** Raman spectra of 1×10^−1^ M, 5×10^−2^ M, 1×10^−2^ M and 1×10^−3^ M acridine molecules from uncoated Ag NRs.
